# On the Use of Emetics in Pulmonary Consumptions

**Published:** 1808-04

**Authors:** 

**Affiliations:** York Street, Dublin


### *On the Use of Emetics in Pulmonary Consumptions.* By Dr. SHARKEY, of York Street, Dublin.

HAVING directed much of my attention to the treatment of Pulmonary Consumption, since the commencement of my practice in physic, I beg you will lay before the public the following cases, which I had noted, not with a view to their publication at present; and which I have selected, not from any peculiarity of the progress of the disease, or of the treatment pursued, but as instances of the result of a plan which I have followed with very considerable success.

The limits of your publication would not allow me (were I even disposed for a theoretical display) to enumerate the physiological reasons, which induced me to consider this as the most rational treatment, in the cure of a disease so deplorable, and which commits such extensive ravages in these countries, as Pulmonary Consumption. Suffice it to say, that I was confirmed in it by the opinions of the first practitioners of the day, though not particularly directed to the end which I have proposed to myself, but thrown out as general physical truths.

It is a melancholy fact to state, that it appears by the London bills of mortality, that 40,000 pesons die annually of pulmonary consumption within the city; and Willan asserts,* that if you except the deaths by small-pox, and those of infants under the age of two years, that the fatality of pulmonary complaints is to the fatality of all others taken together, in the proportion of 5,264 to 8,680; which proportion may undoubtedly be extended to this metropolis.

I was the more inclined to give a fair trial to my plan, after the failure of that of Beddoes, of Pears, &c. and of myrrh, digitalis, lichen islandicus, &c. &c. I do not mean to assert, that I have discovered a new mode of treatment, I only recommend the giving a full and fair trial to a mode which has been adopted from time to time by many practitioners; but I believe very seldom with a sufficient degree of perseverance, without which it will be of no avail: nor do I mean to assert, that it is such a catholicon as to exclude other adjuvants, which I have upon all occasions resorted to, and which the prudent practitioner must adopt as occasion may require, for the relief of urgent incidental symptoms; it is unnecessary for me to add, that there are stages of the disease perfectly hopeless, in which we must not expect to arrest the decay of nature, but suffer her to sink undisturbed; yet I will confidently assert, that, in its incipient stages, nay, before extreme debility and emaciation have taken place, the judicious use of emetics, steadily

* Vide Willan on Diseases in London, particularly in the years 1796, 7, 8, 9, and 1800, page 86.

dily persevered in, will completely eradicate the disease, in a large proportion of cases, unless some untoward disposition be present; such as mal-conformation of thorax, &c. or erosion of the larger blood-vessels of the lungs by vomica, which occurs much more seldom than is generally supposed, but which, if present, would induce a fatal hæmorrhage.

It has been objected to this plan that the constant use of emetics has impaired the stomach, and that the action of vomiting is dangerous, where there exists already actual, or a disposition to hæmoptysis, but I have found the reverse to be the fact; I have uniformly found the appetite improved under their use, at least not impaired, and hæmoptysis removed ; and its removal may be easily reconciled to the principles of pathology ; in no one case have I found an hæmorrhagy to ensue from using emetics : yet I do not deny the possibility of such an event, but I can say that I never met an instance of it; I find the same observation made by many eminent practitioners whom I have consulted ; I believe that fatal hæmorrhagy has very rarely taken place from the bursting of a blood-vessel in the lungs, unless from erosion as above stated, or where there is universal plethora, which cannot be a concomitant of phthisis pulmonalis.*

General Masters, who had been subject to dyspnœa and consumption in his youth, was shot through the lungs, by which he was cured, and lived to an advanced age. Garrick was subject to profuse hæmoptysis, yet lived to a very advanced age; a gentleman who had the care of a military hospital in Belfast, during the rebellion of 1798, assured me, that of 22 persons who had been wounded through the lungs, and were under his care, not one died nor fell into pulmonary consumption: these cases tend to

* No author whom I have consulted has set down the sudden accumulation of fat as an occasional cause of pulmonary consumption, yet I have noted one case in which it appeared to be so; a young lady who lived on George's Quay, about the age of 19, who had been perfectly healthy hitherto, made a visit to some friends in England, and after returning home, became suddenly fat; soon after which she was seized with marked symptoms of phthisis, without any known hereditary predisposition, or exciting cause, and died under the disease, exquisitely characterized, in three weeks. Indeed Celsus seems to have understood this, though his expressions are too general; he says, " ergo si plenor aliquis et speciosior et coloratior factus, suspecta bona sua habere debet, quæ quia neque in eadem subsistere, neque ultra progredi possunt, fere retro, quasi ruinâ quadam revolvuntur." Vid. Cels. de Med. Lib. 2. Chap. 2.

prove, prove, that partial injuries to the lungs, however severe, do not generally produce phthisis.

If from the observations here offered, even a single individual shall be rescued from disease and certain death, I shall be abundantly gratified.

---

Mrs. L's case, age about 25 ; sanguine temperament.

May 14th, 1802. About the beginning of the present month, having put off a flannel petticoat which she had been accustomed to wear, was seized with severe cough, difficult expectoration, shiverings succeeded by great heat. Before this attack she had for some time laboured under a dejected mind, so that she was sometimes obliged to take a glass of wine to rouse her; she often felt as if the floor upon which she stood, or the bed upon which she lay, had suddenly sunk ; now complains of troublesome cough, difficult purulent expectoration, difficulty of breathing, especially at night ; cannot lie in the horizontal posture ; pulse 136; bowels costive ; catamenia heretofore regular. Ordered 15 grains of ipecac. to be taken every second day, as an emetic ; also a mixture with syrup of squills and camphorated tinct. of op. and a fœtid pill at bed time.

15th, Dyspnœa relieved after the emetic ; cough severe, with flying pains through the thorax ; pulse 136, bowels costive. Applicetur emp. vesic sterno ; intermit. pil. fœtida, continuentur cætera.

16th, Has not yet taken her emetic ; pulse 144 ; bowels costive ; no sleep ; pains of thorax relieved ; dyspnœa increased. Six ounces of blood were taken from her arm in my presence, after which the pulse were 132 ; had profuse perspirations in the night. Ordered a pill with calomel and scammony, and a blister between the scapulæ.

18th, Took the emetic, after which the dyspnœa was relieved, but it returned during the night with sense of suffocation ; bowels opened by the pill ; profuse perspiration, especially about the head and neck ; cough and expectoration as before ; face greatly flushed, especially in the evening.

19th, Expectoration of purulent matter more free ; no sleep ; bowels costive ; pulse 120 ; some appetite ; debility increased. Let her have boiled turnips and light mutton broth ; also let her use for common drink, a mixture of equal parts of buttermilk and new milk, with one-third of spring water, kept in a bottle near the fire, and shaken often often until cracked. This preparation of milk has been recommended to me by Dr. Stokes. Took her emetic.

21st, Cheeks greatly flushed towards the evening; general copious perspiration towards morning; cough less troublesome; expectoration more free. Continues the milk diet. Ordered to Ranelagh. Pulse as before. Continue.

23d, Took her emetic, which she says always relieves her; cough less troublesome; strength improved. Continues her pectoral mixture and milk diet as before; sleeps little.

24th, Was up and walked about her room, but complained much of debility; night perspiration diminished; face less flushed; pulse 108. Says she feels as if she could sleep.

27th, Had a return of dyspnœa in the night, which was relieved after the emetic; cough more troublesome; expectoration more difficult; bowels loose from eating oranges, but otherwise seems better. Ordered mistura cretacea—pulse 105.

28th, Bowels relieved; breathing free; expectoration less purulent; appetite improved; continue as before; yawns much.*

June 15th, Has continued her emetics every second day; cough almost gone; night sweats light; sleep natural; appetite improved; strength restored, and in all things better. To take an emetic once a week.

Aug. 1st, Appears in perfect health.

"The Professor of the practice of physic in our University, from whose accurate observations I have derived much important information on this as well as all other occasions upon which I had an opportunity of consulting him, saw this lady with me on the 17th of June, and pronounced her case to be highly dangerous.

I should have observed that she was sometimes delirious, though delirium seldom accompanies hectic fever; I have at present under my care, a patient in a very advanced stage of pulmonary consumption, who is occasionally delirious.

The case of A. T. aged about 35, melancholic temperament, March 31st, 1803.

Has been for several months past troubled with dysp-

* I have observed that yawning is a symptom of returning health in this disease.

næa upon the slightest exercise, with severe cough, and flying stitches through the region of the thorax; perspires much at night, especially about the head and neck; expectoration difficult of yellow matter; cannot lie with ease in the horizontal posture, nor on either side; he some time ago went to the county of Wicklow, by which his health was much improved; but a week ago, having gone out on a wet day, all his symptoms were aggravated, and a great degree of debility supervened; now complains of very troublesome cough at night; much dyspnœa in the horizontal posture; acute pain at scrobiculus cordis, impeding inspiration; pulse 120; face flushed much towards evening. Ordered an emetic of ipecacuan, as in the case of Mrs. L——, also a purgative, and the following pectoral mixture.

℞ Sperm. ceti scrupulos duos, Vitelli ovi recent. dimidium tere simul et adde, aquæ pimento uncias duas, aquæ font. uncias sex. Tinct. op. camphor. unciam, Syrup. scill. semunciam, M. capiat cochlear. amp. urgente tussi.

A blister to be applied to the scrobiculus cordis.

April 4th, Pain relieved by the blister, but complains of a very acute pain in the right side, impeding respiration; has considerable emaciation; ordered venæsectio ad uncias quatuor, and a blister to the side; also emetics as above every third day. Milk diet and a draught of porter occasionally; oysters; forbidden animal food in general.

30th, Continues the emetics regularly; can now lie freely on the left side; respires easily in the horizontal posture; strength greatly recruited and is in good spirits; cannot yet lie with ease on the right side; pulse 90; bowels natural; perspires a little every night, but 'tis general; continues his milk diet; eats a little beef steak at times; his hair falls off readily; had disposition to diarrhœa.

May 4th, Last night he threw up by the emetic some brownish bloody stuff, apparently mixed with pus; he said he felt as if a lump was gathering in his chest for some days before; now can lie with tolerable ease on the right side, he has even slept upon it; eats a little steak; I permitted him to eat oysters, but he got tired of them in a week; pulse 90, of moderate strength; does not now flush in the face; bowels free, but no disposition to diarrhœa.

12th, Continued his emetic regularly, till on the 8th his expectoration was bloody, in consequence of which he discontinued them; I ordered the emetics to be taken on the 9th, since when he has not spit any blood; he has this day gone out in a carriage, and feels greatly recruited in strength; strength; appetite good; continues his milk diet and bit of steak occasionally, and a drink of porter; coughs very little, sleeps well—I ordered an emetic once a week only.

May 23d, free from complaint.

This patient went to live at Enniskillen at my instance, having lived in a very unhealthy part of this town; he lived in the country during three years and was very healthy, but made a visit to Dublin last year, and having been exposed to rain in town, and treating himself negligently, he got pneumonia and died in four days.

The case of Mr. G———, aged about 35, sanguine temperament, a dealer in horses. February 1806.

Some years ago was exposed to rain, and in consequence was seized with the ordinary symptoms of catarrh, which became much more severe within the last fortnight; now complains of great dyspnœa; flying pains in his chest, heavy night sweats, difficult purulent expectoration, hectic flushes, great emaciation and debility; has been blooded by an apothecary about a week ago, since when he thinks his debility greatly increased; at present is unable to get out of bed; pulse 122; has never had spitting of blood, but has undergone heavy salivations for the cure of lues, of which he appears at present well; cannot lie in the horizontal posture, nor upon the left side, but rests supported by bolsters; ordered an emetic as above every second day, also the pectoral mixture and milk diet; he continued in this course for a month, after which he could walk about a little, his strength being somewhat recruited; stitches gone, little night sweats or hectic flushings; cough almost gone; expectoration free, of whitish well digested matter; recovered his flesh; he continued to mend for six or seven weeks, when he insisted on taking a walk round the green with his wife on a thick hazy day; he had only gone the length of one side of the square, when he was suddenly seized with dyspnœa and great debility, so that he was scarcely able to support himself home; his cough and other phthisical symptoms increased rapidly; I was called to see him three days before his death, when I found that colliquative diarrhœa had taken place, and the facies hippocratica was distinctly marked; stomach insensible to an emetic; had also raucedo to such a degree for several days as that he could not utter his words so as to be heard, without a great effort; after which symptom I never saw any patient recover, having observed it in six cases, to all of which except one I had been called in the last stage of the

 disease; disease: the other case was that of a young girl, in whom it arose during my treatment of her; before the accession of the disease, she laboured under amenorrhœa; she complained of too great fatigue from the emetics, and did not persevere in their use.

March 1806. The case of Miss S—— C——, age about twelve, melancholic temperament.

Has been complaining for several weeks of great dyspnœa upon the slightest exercise, especially on going up stairs; pains in her chest, considerable emaciation, short dry cough, hectic flushes in her cheeks in the evenings, little appetite; vessels of the adnata bloodless; much debility; no night sweats. She continued the above emetic plan every third day for six weeks, when all her symptoms of disease had disappeared, and her appetite greatly improved; after which I put her upon a course of the extract of Peruvian bark for three weeks, since which she has remained perfectly healthy, without any symptom of a return.

